# Variations in Magnesium Concentration Are Associated with Increased Mortality: Study in an Unselected Population of Hospitalized Patients

**DOI:** 10.3390/nu12061836

**Published:** 2020-06-19

**Authors:** Justyna Malinowska, Milena Małecka, Olga Ciepiela

**Affiliations:** 1 Students Scientific Group of Laboratory Medicine, Medical University of Warsaw, 02-097 Warsaw, Poland; malinowska_justyna@interia.pl; 2 Department of Laboratory Medicine, Medical University of Warsaw, 02-097 Warsaw, Poland; milena.malecka@wum.edu.pl

**Keywords:** cut-off value, hypomagnesemia, hospitalization, mortality, serum magnesium

## Abstract

Dysmagnesemia is a serious disturbance of microelement homeostasis. The aim of this study was to analyze the distribution of serum magnesium concentrations in hospitalized patients according to gender, age, and result of hospitalization. The study was conducted from February 2018 to January 2019 at the Central Clinical Hospital in Warsaw. Laboratory test results from 20,438 patients were included in this retrospective analysis. When a lower reference value 0.65 mmol/L was applied, hypermagnesemia occurred in 196 patients (1%), hypomagnesemia in 1505 patients (7%), and normomagnesemia in 18,711 patients (92%). At a lower reference value of 0.75 mmol/L, hypomagnesemia was found in 25% and normomagnesemia in 74% of patients. At a lower reference value of 0.85 mmol/L, hypomagnesemia was found in 60% and normomagnesemia in 39% of patients. Either hypo- or hyper-magnesemia was associated with increased risk of in-hospital mortality. This risk is the highest in patients with hypermagnesemia (40.1% of deaths), but also increases inversely with magnesium concentration below 0.85 mmol/L. Serum magnesium concentration was not gender-dependent, and there was a slight positive correlation with age (*p* < 0.0001, r = 0.07). Large fluctuations in serum magnesium level were associated with increased mortality (*p* = 0.0017). The results indicate that dysmagnesemia is associated with severe diseases and generally severe conditions. To avoid misdiagnosis, an increase of a lower cut-off for serum magnesium concentration to at least 0.75 mmol/L is suggested.

## 1. Introduction

Magnesium is one of the most important minerals for maintaining microelement homeostasis. It acts as a cofactor or activator for over 800 enzymes, is essential for neuromuscular conduction, and affects blood glucose level and blood pressure [1,2,3]. Its absorption and excretion are regulated by the kidneys, gut and bones [4]. Magnesium is absorbed primarily in the intestine in an amount of 30–50% of daily intake (~100 mg daily, at a daily recommended intake of 320–420 mg per day for women and men, respectively). There are two pathways in which Mg^2+^ is absorbed: one is a paracellular transport within the small intestine, and the other is a transcellular transport in the cecum and colon [1,4,5,6]. Total storage of magnesium is estimated for 2400 mg, among which 50–60% is stored in bones bound to the hydroxyapatite crystals, 25–30% is stored in muscles, and 20–25% in other soft tissues [5,6,7]. Daily, ~2400 mg of magnesium is filtered by the kidneys’ glomeruli, and up to 99% of this microelement is reabsorbed. This results in daily excretion of magnesium by kidneys in an amount of approximately 100 mg [1,4,5,7]. Unabsorbed magnesium from daily intake is excreted with feces (approximately 270 mg per day) [6]. Mg^2+^ concentration is measured routinely in serum during medical laboratory testing, however it does not provide full information about magnesium homeostasis in the body, since serum concentration constitutes ca. 1% of the total amount of the body’s magnesium [6]. Despite being the second most abundant ion in the cells, its free concentration remains low (up to 1.2 mM). Nevertheless, its role in the cells metabolism is inestimable: bound to polynucleotides, ribosomes, or adenosine triphosphate (ATP), it takes part in nucleotide binding, supports variable enzymatic reaction as a crucial activator or cofactor, allows protein synthesis, and controls cell proliferation [6]. Magnesium homeostasis is connected to calcium and phosphates. Imbalances of those element concentrations result in serious disorders, i.e., arrhythmia, convulsions, and respiratory distress. Renal regulation of its serum concentration occurs through glomerular filtration, tubular reabsorption, or secretion. Under physiological conditions, calcium, phosphates, and magnesium are metabolized by adjusting the amounts excreted in urine to the amount of supply [4].

Hypomagnesemia is reported to be a frequent condition. From the clinical laboratory point of view, hypomagnesemia is defined as decreased serum magnesium concentration below reference ranges. The discussion about reference values for magnesium is still ongoing, since several studies show discrepancies between clinical symptoms of Mg deficiency and the threshold in serum magnesium concentration applied to diagnose magnesium deficiency [8,9,10]. Despite the fact that serum magnesium concentration is routinely used to assess body magnesium status, it has to be underlined that Mg in serum may not correspond with total body magnesium content, since it reflects only 1% of total body stores [2,6,11,12]. Serum magnesium concentration depends, among others, on the daily intake [2,11]. In the NHANES (National Health and Nutrition Examination Survey) results, 45% of the American population was found to be dietary deficient [13], and other studies also point to populational inappropriate magnesium intake [14,15,16]. Magnesium deficiency is associated with enhanced oxidative stress, inflammation, impaired glucose transport, reduced pancreatic insulin secretion, increased insulin resistance, and impaired endothelial function [6,17,18,19]. It is a part of the pathophysiology of type 2 diabetes mellitus, ischemic heart disease, hypertension, dyslipidemia, metabolic syndrome, liver diseases, migraines, and depression [17,19,20,21,22,23,24,25,26,27].

Hypermagnesemia is a less common condition and usually remains undetected. It occurs mostly as a result of chronic kidney disease or the misuse of magnesium-containing supplements or medicines [5]. Despite being a rare condition, extreme hypermagnesemia has serious clinical manifestation and may lead to hypotension, complete heart block, coma, and death [6]. 

As mentioned above, the most commonly used method as an assessment of serum magnesium concentration is a total Mg concentration assessment, which does not provide full information about body magnesium status. Approximately 2% of clinical laboratories offer measurement of ionized magnesium, which represents 55–70% of total magnesium and is biologically active [5,11]. Twenty-four-hour excretion of magnesium in urine gives reliable information about Mg metabolism but should be very carefully used in patients with renal disorders. Insufficient glomerular filtration rate would falsify results of magnesium turnover [5,7,11]. The aforementioned method is particularly useful in assessment of magnesium retention during oral or parenteral loading tests. Decreased excretion of Mg with urine suggests increased magnesium embedding to the bones, which might suggest its depletion. On the other hand, excretion exceeding 60% of magnesium load with the highest probability excludes Mg deficiency [5]. Other methods, which are not routinely and commonly used, are assessment of red blood cell magnesium concentration, soft and hard tissue tests for magnesium content, or isotope studies [5,11].

The aim of this study was to assess the frequency of total serum magnesium imbalance among unselected subjects from inpatient departments using different lower reference values (0.65, 0.75, and 0.85 mmol/L) and its association with in-hospital mortality. 

## 2. Materials and Methods 

We performed a retrospective analysis of serum magnesium assessment among 20,438 patients hospitalized between February 2018 and January 2019 in the Central Clinical Hospital of the Medical University of Warsaw. Among all subjects included, there were 11,537 men (56%) and 8901 women (44%). Average mortality among included subjects was estimated for 3.6%, while overall mortality in the corresponding period of time among all hospitalized patients was 2.3%. Mean age was 60.96 ± 16.90 years, median 61 years (18–99 years). Magnesium concentration was tested upon admission. The frequency of magnesium measurement was dependent on patients’ condition and clinical requirements. For the analysis, the first time-measurements and measurements repeated at least 10 times were included. Peripheral blood was taken by venipuncture into tubes with clotting accelerator and left for 30 min for clotting. Then, samples were centrifuged for 10 min at 1500× *g* and magnesium in serum was measured using a Cobas C702 analyzer (Roche). The principle of the method is based on the reaction of magnesium with xylidyl blue in alkaline solution, which produces a purple diazonium salt. Absorbance of the purple product is directly proportional to the magnesium concentration. The measuring range was 0.10–2.0 mmol/L (0.243–4.86 mg/dL), with high precision (coefficient of variation(CV) 0.7–1.3%).

All data were anonymized. The study included analysis of magnesium concentration, age, gender, and outcome of hospitalization (mortality or survival). As a retrospective analysis, this study was conducted according to rules of the Bioethical Committee of the Medical University of Warsaw.

Statistical analysis was performed using Microsoft Office Excel 2019, GraphPad Prism 6.0, and Statsoft Statistica. A nonparametric analysis was performed using the Mann–Whitney test (difference in magnesium concentration between 2 groups), one-way analysis of variance (ANOVA) (difference in magnesium concentration between age groups), the Fisher exact test (sex difference among different magnesium state groups), and the Kruskal–Wallis test (difference in magnesium concentration between 3 groups), where appropriate. The correlation between age and magnesium concentration was estimated with nonparametric Spearman correlation. Variations in magnesium concentration in patients who were tested at least 10 times were calculated as a delta (∆) between the lowest and the highest serum magnesium concentration during one hospitalization. Normality of distribution of the results was assessed using Shapiro–Wilk and Kolmogorov–Smirnov tests. Results were considered statistically significant at *p* < 0.05.

## 3. Results

The mean serum magnesium concentration was 0.82 ± 0.13 mmol/L. According to different lower reference values of serum magnesium that are recommended in the literature [8,9,11,12], analyses were performed separately for cut-offs of 0.65, 0.75, and 0.85 mmol/L.

### 3.1. Reference Valuesof 0.65–1.2 mmol/L

Hypomagnesemia (Mg < 0.65 mmol/L) was found in 1505 subjects (7%), among which 840 were men (56%) and 665 were women (44%). Patients with hypomagnesemia had a mean Mg concentration of 0.57 ± 0.06 mmol/L.

Normomagnesemia (Mg 0.65–1.2 mmol/L) was found in 18,711 (92%) subjects, among which 10,580 (57%) were men and 8131 (43%) were women. Mean Mg concentration in the normomagnesemic group was 0.83 ± 0.1 mmol/L.

**Figure 1:**
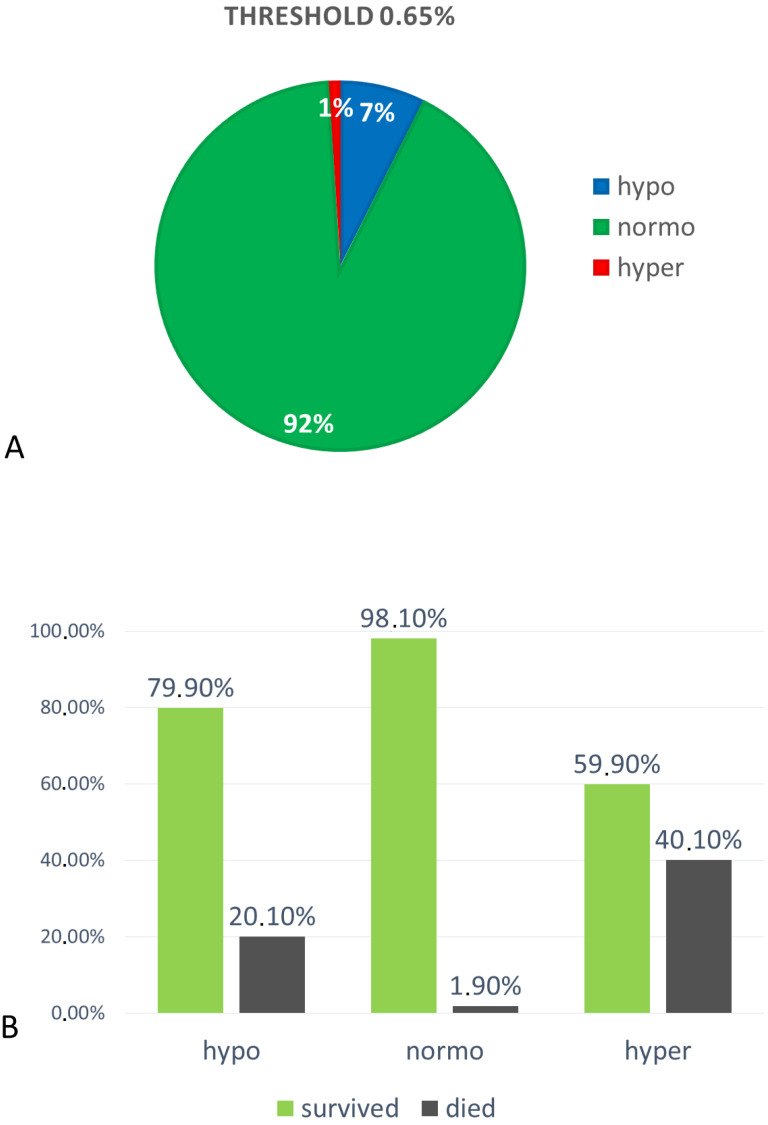
(**A**) Percentages of subjects assigned to hypo-, normo- and hyper-magnesemia groups at reference values of 0.65–1.2 mmol/L, and (**B**) in-hospital mortality ratio among separated groups. The difference between death ratio is statistically significant (Chi-square test), *p* < 0.0001.

Hypermagnesemia (Mg >1.2 mmol/L) was found in 196 subjects (1%), among which 105 (54%) were men and 91 (46%) were women. Mean Mg concentration in this group was 1.36 ± 0.22 mmol/L. All three groups differed significantly in serum magnesium concentration, *p* < 0.0001 (the Kruskal–Wallis test) (Figure 1A).

The highest ratio of in-hospital mortality was found in the group of hypermagnesemic subjects (40.1%). In the group of hypomagnesemic patients, in-hospital mortality was significantly lower (20.1%) than in hypermagnesemic, but also significantly higher than in the normomagnesemic group (1.9%), *p* < 0.0001 (Chi-square test) (Figure 1B).

### 3.2. Reference Values 0.75–1.2 mmol/L

Hypomagnesemia (Mg < 0.75 mmol/L) was found in 5109 subjects (25%), among which 2878 were men (56%) and 2231 were women (44%). Patients with hypomagnesemia had a mean Mg concentration of 0.66 ± 0.07 mmol/L.

Normomagnesemia (Mg 0.75–1.2 mmol/L) was found in 15,183 (74%) subjects, among which 8553 (56%) were men and 6630 (44%) were women. Mean Mg concentration in the normomagnesemic group was 0.86 ± 0.08 mmol/L.

**Figure 2:**
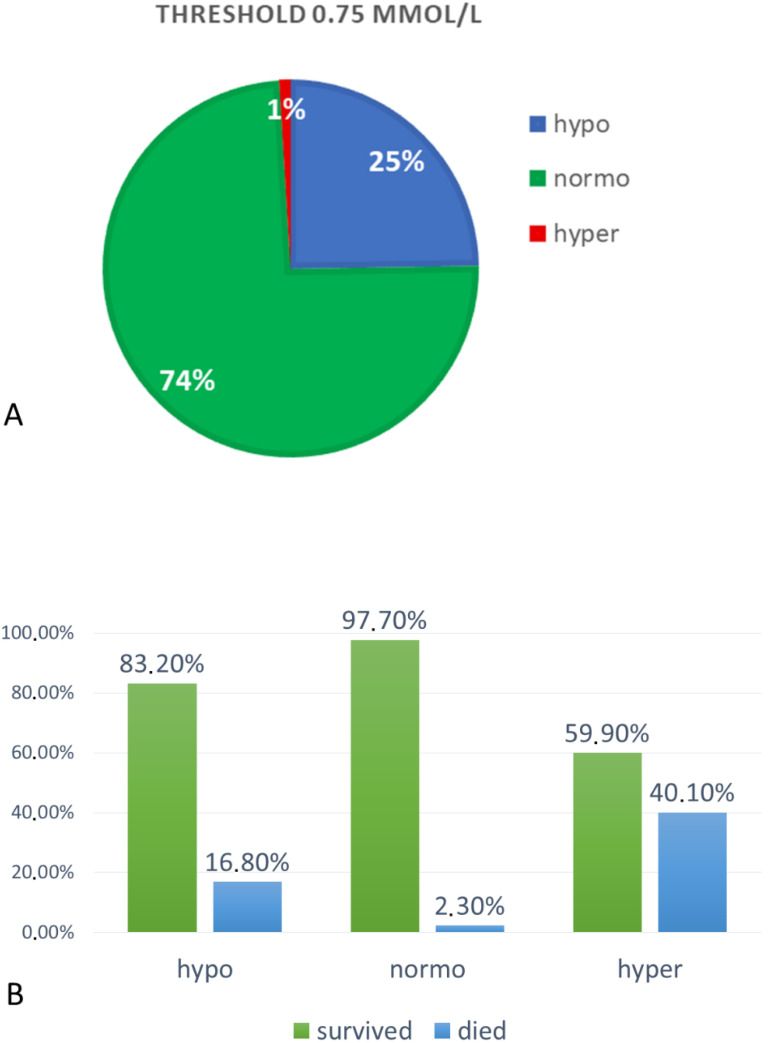
(**A**) Percentages of subjects assigned to hypo-, normo- and hyper-magnesemia groups at reference values of 0.75–1.2 mmol/L, and (**B**) in-hospital mortality ratio among separated groups. The difference between death ratio is statistically significant (Chi-square test), *p* < 0.0001.

Hypermagnesemia (Mg >1.2 mmol/L) was found in 196 subjects (1%), among which 105 (54%) were men and 91 (46%) were women. Mean Mg concentration in this group was 1.36 ± 0.22 mmol/L. All three groups differed significantly in serum magnesium concentration, *p* < 0.0001 (Figure 2A). 

In the group of hypomagnesemic patients, in-hospital mortality was significantly lower (16.8%) than in hypermagnesemic (40.1%), but also significantly higher than in the normomagnesemic group (2.3%), *p* < 0.0001 (Chi-square test) (Figure 2B).

### 3.3. Reference Values 0.85–1.2 mmol/L

Hypomagnesemia (Mg < 0.85 mmol/L) was found in 12,177 subjects (60%), among which 840 were men (56%) and 665 were women (44%). Patients with hypomagnesemia had a mean Mg concentration of 0.74 ± 0.08 mmol/L.

Normomagnesemia (Mg 0.85–1.2 mmol/L) was found in 8064 (39%) subjects, among which 4596 (57%) were men and 3468 (43%) were women. Mean Mg concentration in the normomagnesemic group was 0.92 ± 0.07 mmol/L.

**Figure 3:**
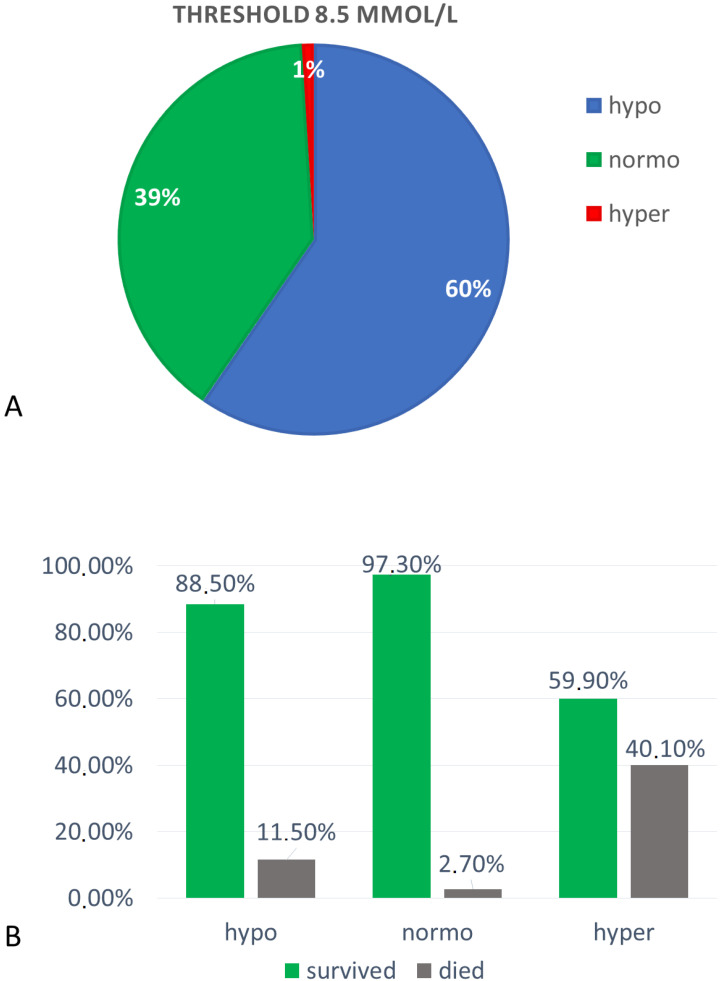
(**A**) Percentages of subjects assigned to hypo-, normo- and hyper-magnesemia groups at reference values of 0.85–1.2 mmol/L, and (**B**) in-hospital mortality ratio among separated groups. The difference between death ratio is statistically significant (Chi-square test), *p* < 0.0001.

Hypermagnesemia (Mg >1.2 mmol/L) was found in 196 subjects (1%), among which 105 (54%) were men and 91 (46%) were women. Mean Mg concentration in this group was 1.36 ± 0.22 mmol/L. All three groups differed significantly in serum magnesium concentration, *p* < 0.0001 (Figure 3A). 

In the group of hypomagnesemic patients, in-hospital mortality was significantly lower (11.5%) than in hypermagnesemic (40.1%), but also significantly higher than in the normomagnesemic group (2.7%), *p* < 0.0001 (Chi-square test) (Figure 3B).

**Figure 4:**
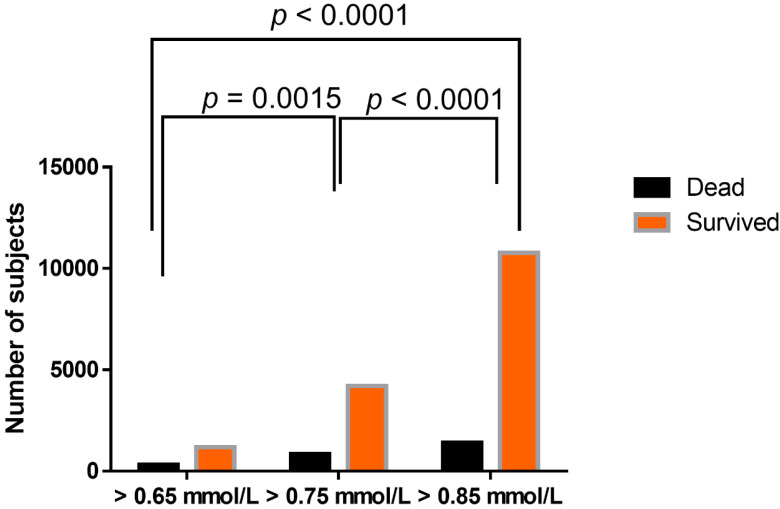
Differences in in-hospital mortality ratio between hypomagnesemic groups in dependence on lower reference value applied. Analysis was performed using Chi-square test, *p* < 0.0001.

There was a statistically significant difference in in-hospital mortality between hypomagnesemic groups, when using different lower reference values of serum magnesium (0.65 mmol/L, 0.75 mmol/L, and 0.85 mmol/L), with the highest ratio of mortality in the group with the lowest reference value applied (Figure 4).

There was no difference in magnesium concentration between men and women, either in all enrolled subjects, or in hypo-, normo-, and hyper-magnesemia groups at all reference values applied, *p* > 0.05 (the Mann–Whitney test). 

**Figure 5:**
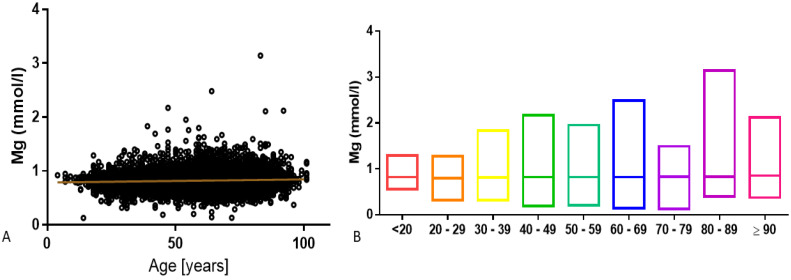
(**A**) Magnesium concentration showed a slight positive correlation with patient age (r = 0.07, *p* < 0.0001), assessed with nonparametric Spearman correlation. (**B**) Median and minimum/maximum values in different age groups.

There was a slight positive correlation between magnesium concentration and age of studied subjects (r = 0.07, *p* < 0.0001) (Figure 5A). However, differences in magnesium concentration were found between different analyzed aged groups (Figure 5B and Table 1)

**Figure 6:**
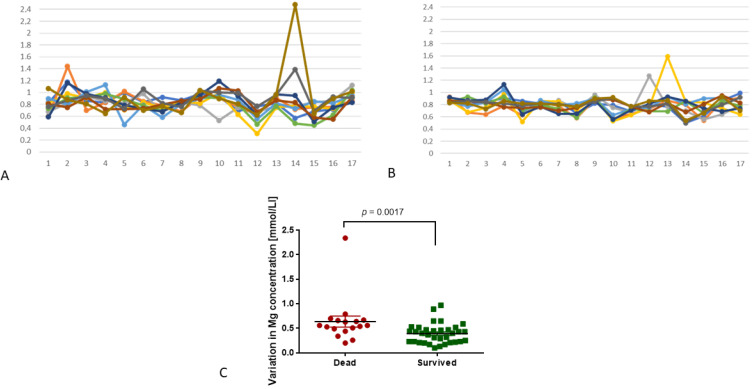
Variation in magnesium concentration in group of patients who were examined at least 10 times and whose hospitalization resulted in (**A**) death or (**B**) survival. Figures A and B present variations in magnesium concentration of ten subjects from each group, in which these fluctuations were expressed the most. The value of magnesium variation was expressed as a difference between the lowest and the highest serum Mg concentration during hospitalization. (**C**) The difference between these two groups assessed with Mann–Whitney test (death, *n* = 17 and survival, *n* = 35) was statistically significant (*p* = 0.0017).

In the study, we also analyzed variation in magnesium concentration in patients who were examined more than 10 times during one hospitalization. This group (*n* = 52) was divided into two subgroups, depending on the outcome of hospitalization: death (*n* = 17, 33%) or survival (*n* = 35, 67%). The variation in magnesium concentration was expressed as a difference between the lowest and highest serum magnesium concentration during one hospitalization (∆). In the group of patients with in-hospital mortality, variations in serum magnesium concentration were higher than in the group of subjects who survived (0.64 ± 0.47 mmol/L vs 0.39 ± 0.2 mmol/L; *p* = 0.0017; Figure 6). We also found that the highest instability in magnesium concentration in the group of subjects with in-hospital mortality was associated with hematological malignancies (0.94 ± 0.79 mmol/L, *n* = 5), septic shock (0.6 ± 0.1 mmol/L, *n* = 2), and multi-organ failure (0.6 ± 0.16 mmol/L, *n* = 4). In the group of patients who survived, magnesium variations were as follows: hematological malignancies 0.44 ± 0.22 mmol/L (*n* = 14, *p* = 0.03), septic shock 0.69 ± 0.15 mmol/L (*n* = 4, *p* = 0.53), and multi-organ failure 0.40 ± 0.13 mmol/L (*n* = 5, *p* = 0.19).

Based on different lower reference values for magnesium concentration in serum, we analyzed how many patients from the group which was tested at least 10 times were hypo-, normo- or hyper-magnesemic upon hospital admission. When setting the reference value for 0.65 mmol/L, there were 44 normomagnesemic and 8 hypomagnesemic patients, among which there was only 1 patient with hypomagnesemia who died during hospitalization (16 were normomagnesemic upon admission). If the lower reference value is set to 0.75 mmol/L, among these 52 subjects, there were 17 hypomagnesemic and 35 normomagnesemic; moreover, 4 hypomagnesemic patients did not survive hospitalization (and 13 normomagnesemic). If we use 0.85 mmol/L as a lower reference value, among 52 patients who were tested at least 10 times, there were 32 hypomagnesemic and 20 normomagnesemic upon admission. Ten patients with hypomagnesemia and seven with normomagnesemia died. None of the patients who were tested at least 10 times had hypermagnesemia upon submission (Table 2).

## 4. Discussion

In the present study, disturbances in magnesium homeostasis were assessed with regard to different lower cut-offs for reference values of serum magnesium concentration: 0.65 mmol/L (applied in the authors’ clinical laboratory), 0.75 mmol/L, and 0.85 mmol/L. At the lowest cut-off value, normomagnesemia was found in 92% of patients, hypomagnesemia was found in 7%, and hypermagnesemia in 1% of studied cases. An increase of the lower reference value for serum magnesium allowed to increase hypomagnesemia frequency to 25% and 60% respectively, and decrease normomagnesemia to 74% and 39%, respectively.

Laboratory relevant hypomagnesemia is recognized when total serum magnesium concentration is below the lower reference value. However, discussion concerning an adequate cut-off for normomagnesemia has been going on since the end of the 20th century. Wong et al. defined hypomagnesemia when total serum Mg concentration was below 0.6 mmol/L, and this value was obtained after assessment of serum magnesium in 341 individuals [10]. von Ehrlich [8] was considering two cut-off values: 0.70 and 0.75 mmol/L to diagnose hypomagnesemia, and found that approximately 9% of patients with a syndrome of magnesium deficiency would remain omitted in clinical practice if the lower cut-off value would be set for 0.7 mmol/L. Increasing the cut-off to 0.75 mmol/L allows to increase the ratio of appropriately diagnosed patients four times. Other studies suggest setting the lower reference value in a range of 0.65-0.95 mmol/L [12,28,29,30,31,32,33]. Nevertheless, it has to be underlined that magnesium deficiency should be assessed clinically, based on deficiency syndromes, not only based on reference values provided by local laboratories or test manufacturers. Costello et al. underline that clinical magnesium deficiency is accurately diagnosed at a cut-off value of 0.82 mmol/L, with proper urinary magnesium excretion (40–80 mg/day) [12]. Liebscher and Liebscher point out that the misdiagnosis ratio of magnesium disturbances could be decreased in a vast majority of patients (99%), if the cut-off value was 0.9 mmol/L. They based their conviction on clinical studies in patients with magnesium disorders, who expressed unspecific symptoms of Mg deficiency attributed to underlying disease [9,11]. 

Here, we found that the hypomagnesemia ratio strictly depends on the cut-off value of serum magnesium concentration. We found hypomagnesemia in 7%, 25%, and 60% of in-patients, using cut-off values of 0.65, 0.75, and 0.85 mmol/L in hospitalized patients. Wong et al., based on a cut-off value of 0.6 mmol/L, recognized hypomagnesemia in 11% of in-patients [10], which is similar to our data and the results of a study performed last year in Italy [29]. Our observation is in contrast to the recent study of Lorenzoni et al., who found that hypomagnesemia at a cut-off value of 0.65 mmol/L is recognized in over 58% of elderly in-patients [28]. We have obtained such a level of hypomagnesemia diagnosis at a cut-off value of 0.85 mmol/L. Our data obtained when a 0.75 mmol/L cut-off value was applied are also in line with Cheungpasitporn et al.’s results, who diagnosed hypomagnesemia (<0.7 mmol/L) in 20.2% of in-patients [31].

Literature data show that increased magnesium concentration in patients with severe conditions is associated with a high risk of mortality. Here, we also confirmed this observation. Similar to an aforementioned study performed in the Mayo Clinic, Rochester, USA [31], we assessed mortality ratio in patients regarding their serum magnesium status. Comparably, we obtained a high mortality rate in subjects of serum magnesium upon admission, with less than 0.75 mmol/L (16.8%) and 0.65 mmol/L (20.1%) [31], but in our study, significantly higher mortality was associated with hypermagnesemia upon admission (40.1%), a group that is much smaller (1%) than hypomagnesemia upon admission (7%, 25%, or 60% depending upon cutoff). Our results are in line with a study by Lorenzoni et al., who assessed a mortality rate in hypermagnesemic subjects of 45% [28]. A higher mortality rate in patients with hypermagnesemia has been also confirmed in other studies [33,34]. This phenomenon may be associated with the influence of magnesium excess on heart muscle function—arrhythmia associated with high magnesium levels may lead to death [34,35,36]. On the other hand, severe conditions may disturb electrolyte homeostasis and induce the release of magnesium from cells, which leads to hypermagnesemia. Moreover, it has been shown that, in general, hypermagnesemia bears a high risk of in-hospital mortality, as well as of 30-day and 12-month mortality [33]. We also observed that mortality in the group of enrolled subjects was higher than overall mortality among all hospitalized subjects in a corresponding period of time. It suggests that, firstly, patients in whom magnesium concentration is assessed upon admission are, in general, in worse condition than others, and secondly, that hypo- (regardless, defined as less than 0.65 or 0.85 mmol/L) and, what is more expressed, hyper-magnesemia, may characterize patients who are more susceptible to in-hospital mortality.

In our study, hypermagnesemia was less frequent than in other studies (1.78–12%) [5,29,30,31]. The differences may be associated with the selection of study group—we did not focus on patients hospitalized due to calcium–phosphate–magnesium disorders, rather we analyzed the general hospitalized population. Regardless of the reference value applied, disturbed magnesium homeostasis is an adverse prognostic factor in in-patient subjects, especially in intensive care units, thus constant and sufficient magnesium concentration might contribute to a decrease of in-hospital mortality [28,37].

Here, we did not observe significant changes in magnesium concentration with patient age—there was only a very slight correlation, which showed that magnesium concentration increases with age. A similar observation was reported by Cheungpasitporn et al. [31]. Interestingly, the mean age of patients with hypomagnesemia was 57.5 years, and with hypermagnesemia, 63 years. Similar observations were reported in other studies, where the mean age with hypomagnesemia was 60 years, and with hypermagnesemia, 65 years [31]. This phenomenon could be associated with inadequate Mg intake in elderly people or decreased kidney function, which appears with age [37]; however, we did not analyze estimated glomerular filtration rate (eGFR) in enrolled subjects and we cannot confirm this observation. Wakai et al. showed that the risk of hypermagnesemia in patients administering magnesium oxide increases in subjects who are ≥68 years old or have decreased eGFR (≤55.4 mL/min), or urea nitrogen of ≥22.4 mg/dL, and their daily administration of magnesium oxide exceeds 1650 mg [38].

Here, we showed that variations in magnesium concentration are associated with in-hospital mortality. This is in line with the observations of Rhee et al., who showed that fluctuations in magnesium concentration in dialyzed patients constitute a risk factor for increased mortality [39]. In our study, the highest variations in magnesium concentration were associated with hematological malignancies, septic shock, and multi-organ disfunction. Literature data show that both hypo- and hyper-magnesemia increase the risk of septic shock in patients who are recognized with systemic inflammatory response syndrome [40]. On the other hand, magnesium was not found as a marker that could help to predict organ failure in critically ill adult patients [41]. Here, we also found differences in magnesium concentration in a group of patients who suffered from different hematological malignancies with different hospitalization outcomes. Significantly higher values were found in subjects with in-hospital mortality. Variation in magnesium concentrations in patients suffering from acute leukemias have already been reported [42] and may be associated with either tumor lysis syndrome triggered by chemotherapy or improper parenteral nutrition. Regardless of etiology, variations in magnesium concentration in these patients may lead to increased mortality due to arrythmia and cardiotoxicity [43].

## 5. Conclusions

To conclude, it has been shown that disturbances in magnesium homeostasis accompany systemic diseases and severe conditions [39,40,43]. Either hypo- or hyper-magnesemia are associated with increased risk of mortality, and, for this reason, primarily, hypermagnesemia should be treated as a laboratory biomarker of critical value. Despite local laboratory reference values for serum magnesium concentration, its deficiency should always be diagnosed based on clinical symptoms. To avoid misdiagnosis, the increase of a lower cut-off value is suggested. High variation in magnesium levels during hospitalization may be associated with increased in-hospital mortality [40,42]; thus, magnesium levels should be routinely tested in hospitalized subjects.

## Figures and Tables

**Table 1:** Statistical differences in median magnesium concentration among studied aged groups (Kruskal–Wallis test).

Compared Aged Groups	Median Mg Values	*p*-Value
<20 vs ≥90	0.82 vs 0.85	<0.01
20–29 vs 40–49	0.80 vs 0.82	<0.001
20–29 vs 50–59	0.80 vs 0.82	<0.0001
20–29 vs 60–69	0.80 vs 0.82	<0.0001
20–29 vs 70–79	0.80 vs 0.83	<0.0001
20–29 vs 80–89	0.80 vs 0.83	<0.0001
20–29 vs ≥90	0.80 vs 0.85	<0.0001
30–39 vs 50–59	0.81 vs 0.82	<0.01
30–39 vs 60–69	0.81 vs 0.82	<0.01
30–39 vs 70–79	0.81 vs 0.83	<0.0001
30–39 vs 80–89	0.81 vs 0.83	<0.0001
30–39 vs ≥90	0.81 vs 0.85	<0.0001
40–49 vs 80–89	0.82 vs 0.83	<0.001
40–49 vs ≥90	0.82 vs 0.85	<0.01
50–59 vs 80–89	0.82 vs 0.83	<0.01
50–59 vs ≥90	0.82 vs 0.85	<0.01
60–69 vs 80–89	0.82 vs 0.83	<0.001
60–69 vs ≥90	0.82 vs 0.85	<0.01
70–79 vs ≥90	0.83 vs 0.85	<0.05

**Table 2:** Number of patients (*n* = 52) tested at least 10 times who died (*n* = 17) or survived hospitalization (*n* = 35) with regard to their magnesium status. Data are separately presented for different lower cut-off for reference magnesium concentration in serum.

	0.65–1.2 mmol/L		0.75–1.2 mmol/L		0.85–1.2 mmol/L	
	Dead *(n*)	Survived (*n*)	Dead (*n*)	Survived (*n*)	Dead (*n*)	Survived (*n*)
**Hypomagnesemia**	1	7	4	13	10	22
**Normomagnesemia**	16	28	13	22	7	13
